# The Role of Lipopolysaccharide-Induced Extracellular Vesicles in Cardiac Cell Death

**DOI:** 10.3390/biology8040069

**Published:** 2019-09-23

**Authors:** Courtnee’ R. Bell, Leandra B. Jones, Brennetta J. Crenshaw, Sanjay Kumar, Glenn C. Rowe, Brian Sims, Gulnaz T. Javan, Qiana L. Matthews

**Affiliations:** 1 Microbiology Program, Department of Biological Sciences, College of Science, Technology, Engineering and Mathematics, Alabama State University, Montgomery, AL 36104, USA; courtneerbell@yahoo.com (C.R.B.); ljones@alasu.edu (L.B.J.); bjcrenshaw0320@gmail.com (B.J.C.); 2 Departments of Pediatrics and Cell, Developmental and Integrative Biology, Division of Neonatology, University of Alabama at Birmingham, Birmingham, AL 35294, USA; skumar@peds.uab.edu (S.K.); bsims@peds.uab.edu (B.S.); 3 Division of Cardiovascular Disease, Department of Medicine, University of Alabama at Birmingham; Birmingham, AL 35294, USA; gcrowe@uab.edu; 4 Forensic Science Program, Physical Sciences Department, Alabama State University, Montgomery, AL 36104, USA; gjavan@alasu.edu; 5 Department of Biological Sciences, College of Science, Technology, Engineering and Mathematics, Alabama State University, Montgomery, AL 36104, USA

**Keywords:** exosome, biogenesis, cardiomyocytes, lipopolysaccharide

## Abstract

Exosomes play a crucial role in the progression of infectious diseases, as exosome release and biogenesis are affected by external factors, such as pathogenic infections. Pyrogens may aide in the progression of diseases by triggering inflammation, endothelial cell injury, and arterial plaque rupture, all of which can lead to acute coronary disease, resulting in cardiac tissue death and the onset of a cardiac event (CE). To better understand the effects of Gram-negative bacterial infections on exosome composition and biogenesis, we examined exosome characteristics after treatment of AC16 human cardiomyocytes with lipopolysaccharide (LPS), which served as a model system for Gram-negative bacterial infection. Using increasing doses (0, 0.1, 1, or 10 µg) of LPS, we showed that treatment with LPS substantially altered the composition of AC16-derived exosomes. Both the relative size and the quantity (particles/mL) of exosomes were decreased significantly at all tested concentrations of LPS treatment compared to the untreated group. In addition, LPS administration reduced the expression of exosomal proteins that are related to exosomal biogenesis. Conversely, we observed an increase in immunomodulators present after LPS administration. This evaluation of the impact of LPS on cardiac cell death and exosome composition will yield new insight into the importance of exosomes in a variety of physiological and pathological processes as it relates to disease progression, diagnosis, and treatment.

## 1. Introduction

Gram-negative bacteria are responsible for millions of nosocomial infections and are of particular interest [1]. These microorganisms are highly efficient at transferring essential genes via quorum sensing, allowing these pathogens to develop mechanisms related to resistance and virulence to further encourage the propagation of the bacteria [2,3,4]. Most Gram-negative bacteria also possess lipopolysaccharide (LPS), an endotoxin that is present in the outer leaflet of the outer membrane of the cell. LPS plays a crucial role in pathogen interactions with the host innate immune system and thus frequently contributes to pathogenesis [2,4,5,6].

Although the precise molecular structure of LPS differs from one bacterium to another, most enzymes and genes related to LPS formulation and transportation were identified in *Escherichia coli* (*E. Coli*) and are shared by the majority of Gram-negative bacteria [2,7]. LPS serves as a model for Gram-negative organisms, such as *E. coli* and *Salmonella sp*., in investigations of the essential components of biogenesis, cell viability, and a myriad of other biological processes [2,6,8]. The assessment of cardiac cell death and exosome biogenesis using LPS as a Gram-negative model for opportunistic pathogens, such as *Pseudomonas aeruginosa,* has not been thorough, despite the urgent need to understand the role of these bacteria in cardiac cell death. 

Cardiovascular disease is the primary cause of roughly one-third of deaths worldwide [9,10]. Given the high mortality rate, new strategies for prevention and intervention of cardiovascular disease are needed. In recent years, studies have focused on understanding the use of extracellular vesicles (EVs) as potential markers of bacterial infection-induced cardiovascular disease. Exosomes, microvesicles, and apoptotic bodies are types of EVs. Although the roles of microvesicles and apoptotic bodies in the cardiovascular system in association with bacterial infections have been examined, exosomes have begun to garner attention in these processes, and future findings may lead to noteworthy research and therapeutic opportunities.

Exosomes are formed from the plasma membrane and the fusion of multi-vesicular bodies and are found in various bodily fluids [7,11]. Exosomes, which range from 30 to 150 nm in size, are EVs that are active in cell-to-cell communication, functioning as mediators of intercellular signaling in the heart [12]. Exosomes are released from several cardiac cell types, including cardiomyocytes, fibroblasts, and endothelial cells [13]. Exosomes transfer proteins and nucleic acids through direct cell contact and also affect cell communication via long-range signaling [14]. Furthermore, interest in exosomes has recently intensified due to the observation that these vesicles play a role in antigen-presenting cell stimulation of the immune responses in vivo [7,15,16]. While previous research has primarily focused on the roles of exosomes in the processes of tumor formation and infectious disease development, our increased understanding of these EVs has fueled an interest in the physiological and pathological functions in a variety of settings, including cardiovascular diseases [17]. In this study, we examined cardiac cell death and exosome composition following the treatment of cardiomyocytes with LPS as a Gram-negative model. These findings will inform our understanding of virulence, pathogenesis, and host responses to infection in the heart, thus potentially leading to better clinical outcomes.

## 2. Materials and Methods

### 2.1. Cell Culture

AC16 human cardiomyocytes (Millipore Sigma) were cultured in Dulbecco’s Modified Eagle’s Medium/Hams F-12 50/50 mix 1 × with L-glutamine (DMEM/F-12; Corning) supplemented with 15% fetal bovine serum (FBS), 1% penicillin-streptomycin, and 4% amphotericin. Cells were maintained at 37 °C with an atmosphere of 5% CO_2_ until the cells reached 70–80% confluency. Cells were then washed with 1 × phosphate-buffered saline (PBS) and removed with 0.05% trypsin, 0.053 mM EDTA, and 1X-sodium bicarbonate. Cells (5 × 10^5^ cells per flask) were then plated in new a T-25 flasks (Corning) in 3 mL of DMEM/F-12 medium. The following day, media were removed, and AC16 cells were washed with sterile 1 × PBS. Following removal of the PBS, exosome-free DMEM/F-12, which was prepared as described above with supplemental 15% exosome-depleted FBS (prepared by System Biosciences), was added.

### 2.2. Cell Treatment with LPS

After replacement of original media with exosome-free DMEM/F-12 the day after plating, cells were treated with 0.1, 1, or 10 µg LPS. For control cells, exosome-free DMEM/F-12 only was used. The cells and LPS were incubated for 48 hours (h) without any further manipulation [18,19,20]. The culture media was collected after 48 h. 

### 2.3. Cell Viability Using Trypan Blue Dye

After harvest, AC16 cells were stained with 0.4% trypan blue solution (Cellgro), and viability was measured using a Countess automated cell counter (Invitrogen).

### 2.4. Exosome Purification and Isolation

Exosomes were collected following 48 h of LPS treatment via filtration and ultracentrifugation. Media was filtered through a 3 mL syringe with a 25 mm syringe filter with a pore size of 0.22 µm. Then, 1 × PBS was added to the media and centrifuged at 32,000 revolutions per minute (rpm) for 70 min in a swinging bucket rotor SW41T1 at 4 °C using a Beckman Coulter Optima L-70K Ultracentrifuge. The supernatant was discarded, and 400–500 µL resuspended exosomes were collected from each sample. Exosome quantitation was performed using the Bradford-Lowry protein quantitation procedure (BioRad).

### 2.5. Exosome Characterization

A Nanosight Tracking Analysis (NTA) NS300 Submicron Particle Imaging System was used to visualize and quantify exosomes from cells treated with LPS. Exosomes were diluted 1:1000, and five independent experiments were analyzed. The mean values for exosome quantity and size measurements were determined. 

### 2.6. Enzyme-Linked Immunosorbent Assay (ELISA)

Exosomes (40 µg) were plated in wells of a 96-well plate. In addition, 50 µL buffer was plated as a control. Then, 100 µL bicarbonate buffer with a pH of 9.5 was added to each well, and plates were incubated at 4 °C overnight. The next day, plates were washed with 1 × PBS with 0.05% Tween 20 three times. After washing, plates were blocked with 5% skim milk in PBS with 0.09% Tween 20 at 4 °C for 1 h. After blocking, primary antibodies were added to each well, and plates were incubated at room temperature (RT) for 2 h or at 4 °C overnight. After incubation, the plates were rewashed as described above, and horseradish peroxidase-conjugated secondary antibody (anti-mouse, 1:1000 or anti-rabbit, 1:1000; Dako) was added to each well for incubation at RT for 2 h or at 4 °C overnight. After incubation, the ELISAs were developed with SigmaFast™ OPD peroxidase substrate (Sigma Aldrich) in the dark at RT for 30 min. Intensities were determined at 405 nm using a Genemate UniRead 800 microplate reader.

### 2.7. Statistical Analysis

Statistical analyses were conducted using the standard functions of Excel and GraphPad Prism (version 5.0,GraphPad Software, San Diego, CA, USA). Mean values were compared using an unpaired two-tailed *t*-test and ANOVA. The error of probability was indicated as * *p* ≤ 0.05, ** *p* ≤ 0.01, *** *p* ≤ 0.001.

## 3. Results

### 3.1. Viability of Cardiomyocytes after LPS Treatment

**Figure 1:**
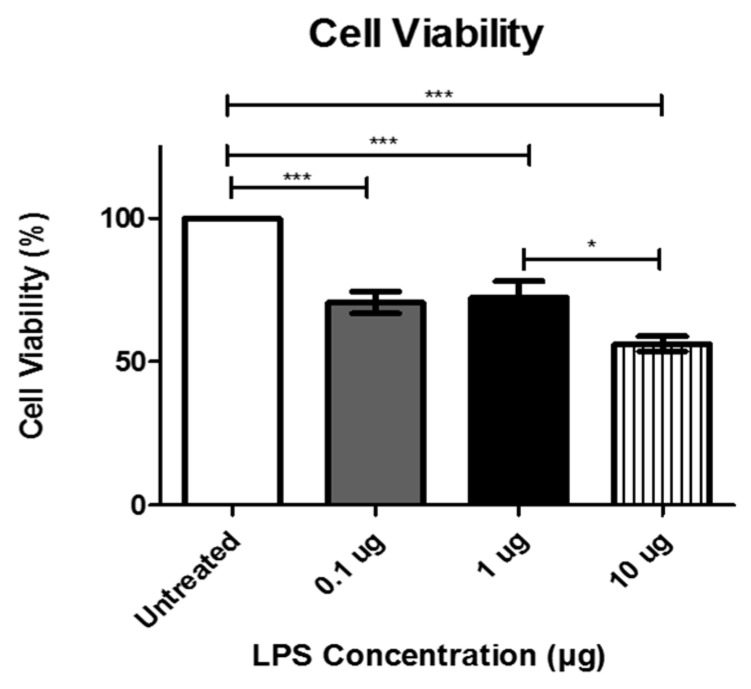
Cell viability following treatment with the indicated concentrations of lipopolysaccharide LPS was determined using MTT assay at 48 h. Data are shown as means ± SEM from a total of five experiments. Significance is indicated by * *p* ≤ 0.05 and *** *p* ≤ 0.001.

To examine the effect of LPS treatment on human cardiomyocytes, AC16 cells were treated with increasing concentrations of LPS, which represent varying levels of bacterial infection [18,19,20]. Cell viability was measured after 48 h of LPS treatment. LPS treatment at 0.1 and 1 µg decreased cell viability to 70%, while treatment with 10 µg of LPS decreased the cell viability to approximately 55% compared to the control group (Figure 1). The effect on cell viability was further examined using the Live/Dead Stain. LPS-treated cells were visibly less dense than the untreated cells [21] confirming that LPS treatment decreased cell viability.

### 3.2. Exosome Characterization after LPS Treatment

**Figure 2:**
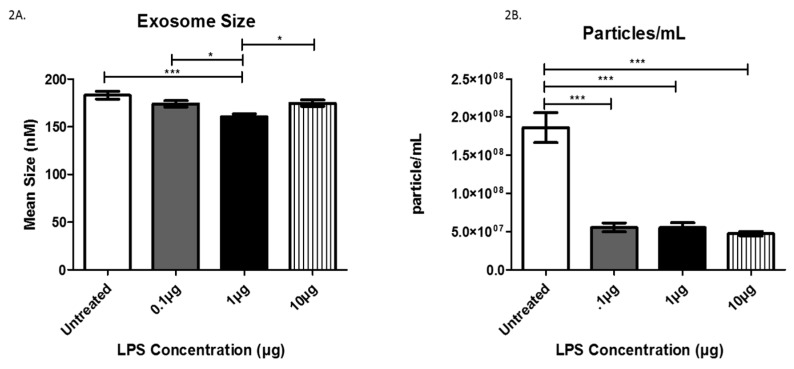
The mean sizes (**A**) and the number of particles/mL (**B**) were determined for AC16-derived exosomes after LPS treatment using Nanosight Tracking Analysis. Data are shown as means ± SEM from a total of five experiments. Significance is indicated by * *p* ≤ 0.05 and *** *p* ≤ 0.001.

The mean exosome size and concentration were determined using NTA (Figure 2), which is software used to track the Brownian motion of individual vesicles. Analysis of LPS-induced exosomes revealed a significant decrease in exosome mean size compared between groups. Specifically, significant decreases from the untreated cells to those treated with 1 µg LPS, from the cells treated with 0.1 µg LPS to those treated with 1 µg LPS, and from the cells treated with 1 µg LPS to those treated with 10 µg LPS (Figure 2A).

Further Nanosight Tracking Analysis (NTA) analysis revealed a significant decrease in the number of exosome particles/mL after LPS treatment (Figure 2B). In the untreated cells, the average number of exosome particles was 1.8 × 10^8^ particles/mL, while cells treated with 0.1 and 1 µg LPS yielded decreased numbers of exosome particles (5.54 × 10^7^/mL). Furthermore, treatment with 10 µg LPS decreased the number of exosomes to 4.73 × 10^7^ particles/mL. These findings indicate AC16 cell exposure to LPS significantly decreases the number of exosomes (particles/mL) at all LPS concentrations tested compared to untreated cells.

### 3.3. Analysis of Exosome-Associated Proteins in LPS-Treated AC16 Cells via ELISA

**Figure 3:**
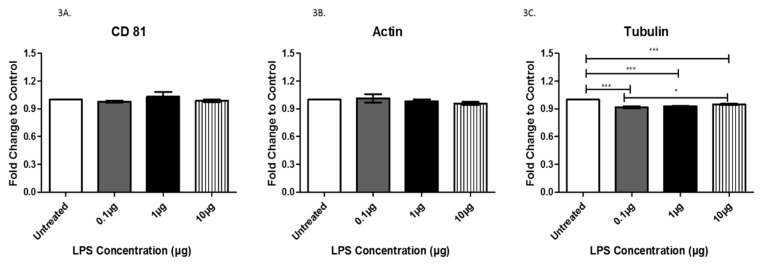
The expression of CD81 (**A**), actin (**B**), and tubulin (**C**) was determined by ELISA in exosomes from AC16 cells that were treated with different concentrations of LPS or left untreated. Data are shown as means ± SEM from a total of five experiments. Significance is indicated by * *p* ≤ 0.05 and *** *p* ≤ 0.001.

Next, we evaluated the expression of proteins associated with exosomes. Specifically, CD81, actin, tubulin, Rab5, Rab27A, Toll-like receptor 4 (TLR 4), tumor necrosis factor-alpha (TNFα), cleaved caspase 3, and cleaved caspase 9 were evaluated to investigate any changes in expression following LPS treatment. CD81 and actin were both expressed in exosomes, as expected (Figure 3A,B). We also evaluated CD9 and CD63 [21], which are both classical exosome markers, but neither were found to be present in our exosomes. Although LPS treatment did not induce any significant changes in the expression of CD81 and actin, the mere presence of the characteristic exosomal tetraspanin CD81 and actin, a protein commonly associated with exosomes, further confirms that we successfully collected and purified exosomes. Additionally, exosomes from cells treated with LPS exhibited a significant decrease in expression of tubulin, a common cytoskeleton-binding protein marker, from the untreated cells to each treated group with a mean difference of +/− 8.3 × 10^−2^ (0.1 μg group), +/−7.3 × 10^−2^ (1 μg group), and +/− 5.2 × 10^−2^ (10 μg group) (Figure 3C). Interestingly, comparison of exosomes from cells treated with 0.1 and 10 µg LPS revealed a significant increase in tubulin expression at the higher LPS dose (Figure 3C).

**Figure 4:**
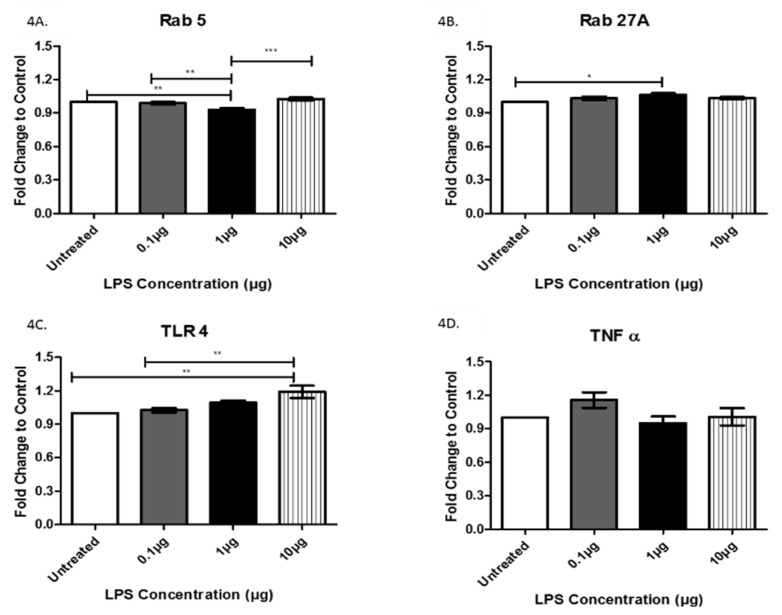
The expression of Rab5 (**A**), Rab27A (**B**), TLR4 (**C**), and TNFα (**D**) was determined by ELISA in exosomes from AC16 cells that were treated with different concentrations of LPS or left untreated. Data are shown as means ± SEM from a total of five experiments. Significance is indicated by * *p* ≤ 0.05, ** *p* ≤ 0.01 and *** *p* ≤ 0.001.

**Figure 5:**
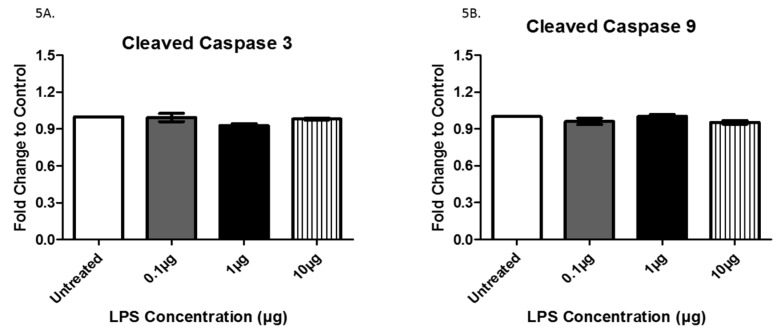
The expression of cleaved caspase 3 (**A**) and 9 (**B**) was determined by ELISA in exosomes from AC16 cells that were treated with different concentrations of LPS or left untreated. Data are shown as means ± SEM from a total of five experiments.

Rab proteins are a type of G protein that function in the regulation of vesicle formation and membrane trafficking. The expression of Rab5, a regulator of vesicle sorting, was significantly decreased in exosomes collected from cells treated with 1 µg LPS compared to untreated controls (+/− 6.9 × 10^−2^). Additionally, Rab5 was also decreased in exosomes from cells treated with 0.1 μg LPS compared to those treated with 1 μg LPS (+/− 5.8 × 10^−2^) and was significantly increased in exosomes from cells treated with 10 μg LPS compared to those treated with 1 μg LPS (+/− 9.4 × 10^−2^) (Figure 4A). Rab27A, which plays an essential role in vesicle trafficking, showed a slight increase in exosomes collected after treatment with 0.1 and 1 µg (+/− 6.0 × 10^−2^) of LPS compared to exosomes from untreated controls (Figure 4B). TLR4 and TNFα, which both play roles in the immune response and inflammation, were expressed in AC16 cell-derived-exosomes (Figure 4C,D). The expression of TLR4 was significantly increased following treatment with 10 μg LPS compared to both the untreated (+/− 1.8 × 10^−2^) and the 0.1 µg (+/− 1.6 × 10^−2^) LPS-treated cells (Figure 4C). Furthermore, TLR4 exhibited a trend of increased expression with increased LPS concentration. In general, the different LPS concentrations did not significantly alter TNFα expression (Figure 4D). The cleaved active forms of caspase 3 and caspase 9, which are both involved in apoptosis mediation, were expressed in AC16 cell-derived-exosomes; however, LPS treatment had no significant effect on their expression levels (Figure 5A,B). While LPS treatment had no significant effect on the protein expression of CD81 (Figure 3A), actin (Figure 3B), TNFα (Figure 4D), caspase 3 (Figure 5A), and caspase 9 (Figure 5B), the expression of these proteins in the purified exosomes all exhibited a unique change following treatment with 1 μg LPS.

## 4. Discussion 

Gram-negative bacterial infections are responsible for millions of infections and deaths worldwide [1]. With an increase in antibiotic resistance in opportunistic bacteria that can invade the heart, and a concomitant decrease in new effective drugs to treat these bacterial infections, there is a pressing need to understand the role of Gram-negative bacteria in cardiac cell death to inform the development of novel treatment strategies. Thus, an examination of the intricate changes that occur in the heart after Gram-negative bacterial infection is necessary to bolster our understanding. Studies performed by Yang et al. 2018, showed that microglia derived EV’s played a role in the propagation of inflammation and cell-to-cell communication in the Central Nervous System (CNS). This, along with other supporting factors, makes EVs, in particular exosomes, attractive targets for cell communication studies [20]. In this study, we evaluated the effects of LPS treatment on exosome biogenesis and composition in AC16 cardiomyocytes. 

The primary causative events of Gram-negative bacterial infections are bacteremia, and septic shock, which are caused by the release of LPS [22]. We selected varying concentrations of LPS for our experiments to correspond to different levels of bacterial infection. Evaluating sub-lethal infection levels will allow for a better understanding of the different immune and cellular responses elicited by these pathogens. In our experiments, treatment with LPS caused a significant decrease in overall cell viability. Analysis of exosome size and numbers revealed that LPS induced a significant decrease in mean exosome size as well as exosome particles/mL (Figure 2A,B). These findings indicate that, as cells are infected with Gram-negative bacteria, the size of the exosomes decreases substantially, corresponding to the observed decrease in cell viability. This decreased cell survival also is mirrored by the decline in the number of exosomes released from the cells. We estimated the exosome particle/cell number at 48 h post-treatment to be 186 exosomes/per cells (control), 79.1 exosomes/per cells (0.1 µg), 76.9 exosomes/per cells (1.0 µg), and 84.5 exosomes/per cell (10 µg). Together, these findings demonstrate that the initiation of cardiomyocyte death by LPS released from Gram-negative bacteria leads to the production of fewer exosomes in response to the infection.

CD81 is a small membrane tetraspanin that is characteristically found on exosomes and is involved in a wide variety of biological functions, such as cell migration, cell proliferation, immune response, and thrombosis [23]. CD81 has also been shown to be induced by oxidative stress, and its enhanced expression appears to play a crucial role in the initial stages of atherosclerotic plaque formation [24]. Although our data did not show a significant increase in CD81 in response to LPS treatment, the presence and the upward trend of CD81 in exosomes in response to LPS treatment was evident. 

The cytoskeleton of cardiac myocytes consists of actin, the intermediate filament, and alpha- and beta-tubulin, which form the microtubules [25]. The cytoskeleton of these microtubules contributes to cell structure, motility, division, and intracellular transport. If altered, microtubules can pose an increased load on cardiomyocytes, ultimately impeding sarcomere motion and promoting cardiac dysfunction and irreversible cardiac cell death [17,26,27]. The observed slight increase of actin in LPS-treated exosomes mirrored the increasing LPS concentrations, indicating a potential marginal change in cell structure, motility, and division. Our data suggest that tubulin is significantly decreased in exosomes following the LPS treatment of AC16 cells, indicating a substantial change in the amount of tubulin expressed in the exosomes and potentially leading to improper microtubule formation as a result of bacterial infection.

Rab5 and Rab27A belong to a family of GTPases that play an essential role in intracellular vesicle transport via control of membrane trafficking between intracellular compartments [28]. Rab5 is ubiquitous and functions in standard steps in the exocytic and endocytic pathways of all mammalian cells. Rab5 is purported to promote the motility of early endosomes on microtubules [29]. Rab27 is more tissue-restricted, suggesting a more specialized function for this GTPase [30]. The LPS-induced decreased expression of Rab5, without similar effects on Rab27A, suggests that the regulatory interactions with the microtubule network may be slowed as a result of bacterial infection.

A previous study conducted by Davidson et al. showed that plasma exosomes protect the hearts of rats from injury both in vitro and in vivo [31]. These studies revealed that heat shock protein 70 (HSP70) on the exosomal surface-induced cardioprotection by stimulating TLR4 on cardiomyocytes. Our data demonstrate that TLR4 is significantly increased by increasing concentrations of LPS, suggesting that exosomes may facilitate some type of protective function against bacterial infection [10,31]. Pro-inflammatory macrophages indirectly exacerbate both atherosclerotic and aortic valve calcification via the secretion of inflammatory cytokines such as TNFα [32], which may explain the upward trend observed for TNFα expression following LPS treatment of cardiomyocytes.

Caspases are a family of proteases that play a role in programmed cell death and inflammation. A study conducted by Park et al. concluded that the release of exosomes is dependent upon caspases, particularly caspase 3 and caspase 9 [33]. In our study, we showed that both cleaved caspase 3 and cleaved caspase 9 expression were not altered after LPS treatment. This observation is interesting as we also found a significant decrease in exosome size and quantity, refuting the notion that exosome production is directly correlated to these particular caspase levels. Although the expression of caspases 3 and 9 are not changed by LPS treatment, these enzymes may still play a role in mediating cell death. Further investigation of cell lysates is warranted in order to examine these relationships more thoroughly.

## 5. Conclusions

In conclusion, we confirmed the presence of exosomes from AC16 cardiac cells and described the relative exosomal changes after the treatment of these cells with LPS. LPS administration significantly decreased AC16 cell viability, exosome quantity, and exosome size. Exosomal proteins were altered by LPS treatment, with a significant decrease in structural and vesicle trafficking proteins. These data illuminate the facilitatory role that exosomes and their constituents play in cardiac cell death after bacterial infection and, therefore, these findings could have clinical implications. In particular, the non- invasive surveillance of exosomes numbers in the form of a blood test could provide information regarding Gram-negative bacterial infections. In future studies in AC16 cells, we will compare the effects of LPS treatment with those induced by infection by some common opportunistic Gram-negative bacteria. Furthermore, these studies shed light on the impact of LPS on cardiac cell death and exosome biogenesis and offer insight into the importance of exosomes in a variety of processes related to cardiovascular disease. Additional investigations will allow us to identify potential exosomal markers for the further identification of pathogen-specific contributions to cardiovascular disease.

## Data Accessibility

The original data will be maintained by the corresponding author. Information pertaining to the data sets will be made available upon written request.
